# Comparative transcriptome analysis of flower bud transition and functional characterization of *EjAGL17* involved in regulating floral initiation in loquat

**DOI:** 10.1371/journal.pone.0239382

**Published:** 2020-10-08

**Authors:** Yan Xia, Baogui Xue, Min Shi, Feng Zhan, Di Wu, Danlong Jing, Shuming Wang, Qigao Guo, Guolu Liang, Qiao He

**Affiliations:** 1 Key Laboratory of Horticulture Science for Southern Mountains Regions of Ministry of Education, College of Horticulture and Landscape Architecture, Southwest University, Beibei, Chongqing, China; 2 Academy of Agricultural Sciences of Southwest University, State Cultivation Base of Crop Stress Biology for Southern Mountainous Land of Southwest University, Beibei, Chongqing, China; Huazhong University of Science and Technology, CHINA

## Abstract

Floral initiation plays a critical role for reproductive success in plants, especially fruit trees. However, little information is known on the mechanism of the initiation in loquat (*Eriobotrya japonica* Lindl.). Here, we used transcriptomic, expression and functional analysis to investigate the candidate genes in floral initiation in loquat. Comparative transcriptome analysis showed differentially expressed genes (DEGs) were mainly enriched in the metabolic pathways of plant hormone signal transduction. The DEGs were mainly involved in the gibberellin, auxin, cytokinin, abscisic acid, salicylic acid and ethylene signaling pathways. Meanwhile, some transcription factors, including *MADS-box* (MCM1, AGAMOUS, DEFICIENS and SRF), *MYB* (Myeloblastosis), *TCP* (TEOSINTE BRANCHED 1, CYCLOIDEA and PCF1), *WOX* (WUSCHEL-related homeobox) and *WRKY* (WRKY DNA-binding protein), were significantly differentially expressed. Among these key DEGs, we confirmed that an *AGL17* ortholog *EjAGL17* was significantly upregulated at the flower bud transition stage. Phylogenetic tree analysis revealed that EjAGL17 was grouped into an AGL17 clade of MADS-box transcription factors. Protein sequence alignment showed that EjAGL17 included a distinctive C-terminal domain. Subcellular localization of *EjAGL17* was found only in the nucleus. Expression levels of *EjAGL17* reached the highest at the development stage of flower bud transition. Moreover, ectopic expression of *EjAGL17* in *Arabidopsis* significantly exhibited early flowering. Our study provides abundant resources of candidate genes for studying the mechanisms underlying the floral initiation in loquat and other Rosaceae species.

## 1. Introduction

Floral initiation is a critical phase for reproductive success in the life cycle of plants [1, 2]. Molecular mechanisms of floral initiation are based primarily on studies in the model plant *Arabidopsis* [2–4]. Flower initiation is controlled by the environmental signals and molecular networks in *Arabidopsis*. Among the molecular networks, floral integrators including *FLOWERING LOCUS T* (*FT*) and *SUPPRESSOR OF OVEREXPRESSION OF CONSTANS 1* (*SOC1*), and transcription factors such as *MADS-box* (MCM1, AGAMOUS, DEFICIENS and SRF) genes play important regulatory roles in floral initiation and flowering time control, providing a fundament for studying gene regulation of flower development in angiosperm [5–13].

Transcriptomics is an effective method for discovering the gene networks and regulatory mechanisms underlying floral initiation in non-model plants [14–16]. For example, in *Rosa chinensis*, comparative transcriptome analysis of the floral transition revealed that differentially expressed genes (DEGs) are mainly involved in the pathways of vernalization, photoperiod and gibberellin (GA) [14]. In *Agropyron cristatum*, transcriptomic analysis of floral transition indicated that DEGs are mainly involved in plant circadian clock pathway and flowering time-associated genes [15]. Comparative transcriptome analysis indicated that DEGs of cytokinin biosynthesis pathway are mainly associated with floral transition in *Dendrobium nobile*. However, global gene expression changes of floral initiation in *Eriobotrya* remain unknown and need further investigated.

Loquat (*Eriobotrya japonica* Lindl.) is a subtropical evergreen species and belongs to the Amygdaloideae subfamily of the Rosaceae [17]. Floral initiation of loquat is a continuous process, which is not interrupted by winter dormancy [18, 19]. In this study, we first investigated the transcriptomic changes in the floral transition of loquat between vegetative apex (VA) and flower bud transition (FBT). The DEGs were mainly involved in the metabolic pathways of hormone signal transduction. Furthermore, we identified that a candidate gene *EjAGL17* was significantly upregulated at the flower bud transition stage and involved in floral initiation. Our results provide abundant gene resources for studying the mechanisms underlying the floral initiation in loquat.

## 2. Materials and methods

### 2.1 Plant material

Flower buds of loquat cultivar ‘Changbai No.1’ at different development stages were provided and collected from the experimental farm of Southwest University in Xiema town (Chongqing, China). The field experiments were conducted under local legislation and permissions. According to our previous study, the development stages included vegetative apex (S1), flower bud transition and flower bud differentiation (S2), elongation of the main axis in a panicle (S3), rapid panicle elongation (S4), visible floral buds (S5), elongation of branches in a panicle (S6), white corollas (S7) and full bloom (S8) [20]. Then, these samples were frozen immediately in liquid nitrogen and stored at -80°C until used.

### 2.2 Morphological analysis of the floral initiation

Morphology of developmental stages was collected and photographed using Canon EOS 6D (Canon Inc., Tokyo, Japan) according to the stages described previously [20]. The buds of loquat at the stage of vegetative apex (VA) and flower bud transition (FBT) were fixed in a FAA solution under vacuum. Samples were dehydrated by a series of ethanol solution, and then decolorized by dimethylbenzene: ethanol (50:50, by vol.) and 100% dimethylbenzene. Then, samples were treated using three changes of 100% paraffin at 60°C and finally embedded. The paraffin-embedded samples were cut to a thickness of 10 μm. After dewaxing, the samples were stained with safranine-fast green solution and photographed using an Observer DP80 microscope (Olympus, Tokyo, Japan).

### 2.3 RNA extraction, library consatruction and sequencing

Total RNA was extracted from VA and FBT samples using the EASYspin Plant RNA Extraction kit (Aidlab, Beijing, China). Three independent biological replicates were performed for each sample. Residual DNA was removed from the total RNA using RNase-free DNase I (Takara, Japan) at 37°C for 20 min. Integrity of RNA was validated by 1% agarose gel and Agilent 2100 Bioanalyzer (Agilent Technologies, Palo Alto, CA, USA). Then, oligo (dT)-attached magnetic beads enriched method was used in purifying mRNA.

The mRNAs were broken into short fragments and synthesized second-strand cDNA. Then, the synthesized cDNA fragments were subjected to end pairing, the addition of a single 'A' nucleotide and ligation with sequencing adapters. The ligation fragments were amplified by PCR, and sequenced using an Illumina HiSeq X-Ten platform (Shanghai Personal Biotechnology Co., Ltd., China). Sequencing raw data are available with an accession number CRA003214 in the Genome Sequence Archive (GSA, https://bigd.big.ac.cn/gsa/).

### 2.4 Assembly and functional annotation

The raw reads containing adapter sequences and low quality bases were removed. Reads containing ambiguous 'N' bases, and fragment less than 50 bp were filtered to generate high quality clean reads. Then, the clean reads were subsequently assembled using Trinity assembly program [21]. Basic Local Alignment Search Tool (BLAST) was used to annotate the function of unique sequences using the databases of the non-redundant (NR) proteins, Swiss-Prot, Gene Ontology (GO) [22], evolutionary genealogy of genes: Non-supervised Orthologous Groups (eggNOG) [23] and Kyoto Encyclopedia of Genes and Genomes (KEGG) [24].

### 2.5 Analysis of differentially expressed genes (DEGs)

Expression of unigenes were calculated by the method of fragments per kb per million from the mapped reads (FPKM) [25]. Differential expression of genes were analyzed using DESeq software [26], and unigenes showing at least a two-fold change and *P-*value ≤ 0.05 were considered to be DEGs. Then, the DEGs were mapped to GO terms and KEGG pathways the method described previously [27, 28].

### 2.6 Validation of RNA-Seq analysis by qRT-PCR

Total RNA was extracted from the collected from VA and FBT. Then, 2 μg RNA of each sample was used for cDNA synthesis using the PrimeScript RT reagent kit (Takara, Japan). For quantitative real-time PCR (qRT-PCR), 1 μL cDNA was used as a template in a 20 μL PCR. Then, qRT-PCR was performed using CFX96 Touch Real-time PCR Detection System (BioRad, USA). The parameters were at 95°C for 5 min, followed by 40 cycles of 95°C for 20 s, 56°C for 20 s, 72°C for 20 s; then, temperature change 1.0°C/s to 95°C for melt curve. The *actin* of loquat was used as a normalization control with the primers qEjactinF and qEjactinR [29]. These primers of subcellular localization and qRT-PCR were shown in S1 Table. Three biological replicates of each sample were performed. The expression levels were calculated using the 2^-ΔΔCT^ method [30].

### 2.7 Isolation of an *AGL17* ortholog in loquat

Total RNA was extracted from flower buds of loquat as described above. The extracted RNA was treated with DNase I (TakaRa, Japan), and the concentrations were detected by UV spectrophotometry (NanoDrop 2000, Thermo Scientific, USA). Then, the DNase I-treated RNA was used for rapid amplification of cDNA ends (RACE). From these key DEGs obtained by RNA-Seq, the primers of an *AGL17* ortholog (*EjAGL17*) were designed from the DN37075_c0_g1 sequence. The 3′ RACE of *EjAGL17* was performed using 3′-full RACE Core Set Version 2.0 kit (Takara, Japan) and 2 μg of the DNase I-treated RNA. First PCR was performed using 3′ RACE Outer Primer and the gene-specific primer 3RAGL17F1. A second PCR was performed using 3′ RACE Inner Primer and 3RAGL17F2. PCR parameters were a 5 min at 95°C denaturation step, followed by 30 cycles of 40 s at 95°C, 40 s annealing at 56°C and 40 s extension at 72°C, with a final step of 10 min at 72°C. The 5′ RACE of *EjAGL17* was carried out using 2 μg of the DNase I-treated RNA and SMARTer RACE 5′/3′ Kit (Takara, Japan). PCR amplification was performed using Universal Primer Mix, the gene-specific primer 5RAGL17R1 and 5RAGL17R2. PCR parameters were 5 cycles at 95°C for 30 s, 72°C for 3 min; 5 cycles of 95°C for 30 s, 70°C for 30 s, 72°C for 3 min; 25 cycles of 95°C for 30 s, 68°C for 30 s and 72°C for 3 min.

2 μg of the DNase I-treated RNA was used for cDNA synthesis using M-MLV reverse transcriptase and oligo(dT)-15 adaptor primer (Takara, Japan). Then, the cDNA sequence integrity of *EjAGL17* was verified using the primers FLAGL17F and FLAGL17R. PCR parameters were at 95°C denaturation step for 5 min, followed by 30 cycles of 95°C for 50 s, 56°C for 50 s and 72°C for 40 s, with a final extension step of 72°C for 10 min. The primer sequences of *EjAGL17* isolation were shown in S2 Table. Finally, PCR products were recycled and cloned into pMD18-T vector (Takara, Japan) and sequenced.

### 2.8 Analyses of sequence alignment and phylogenetic tree

The BLAST analysis of amino acid sequences of EjAGL17 was performed. Multiple AG, AGL17, SOC1, AP1, SEP, AP3, PI and TM6-clade proteins from various eudicots were selected for alignment from the GenBank database. Accession numbers of these clade proteins were selected for alignment from various eudicots and listed in S3 Table. A ClustalW program was used to align amino acid sequences of these proteins including the M, I, K and C domains [31]. A phylogenetic tree was constructed by MEGA 5.0 software [32, 33], and the parameters were the maximum likelihood method, 1000 replicates of bootstrap and Jones–Taylor–Thornton (JTT) model [34, 35].

### 2.9 Subcellular localization and quantitative real-time PCR

Subcellular localization of *EjAGL17* was observed using the modified pCAMBIA 1300 vector and the primers SLEjAGL17F and SLEjAGL17R [36]. Then, the 35S::*EjAGL17-GFP* was constructed and transformed into *Nicotiana benthamiana* leaves using *Agrobacterium*-mediated transient transformation. The vector expressing GFP alone was as a negative control. Green fluorescent protein (GFP) fluorescence signals were observed using a fluorescence microscope Observer DP80 (Olympus, Japan).

Total RNA was extracted from the collected flower buds of eight developmental stages (S1-S8). Then, 2μg RNA of each sample was used for cDNA synthesis as described above. For quantitative real-time PCR (qRT-PCR), the primers of subcellular localization and qRT-PCR were shown in S4 Table.

### 2.10 Vectors construction and *Arabidopsis* transformation

The *EjAGL17* coding sequences was cloned into the expression vector pBI121 (BD Biosciences Clontech, USA) using the restriction enzymes of *Xba* I and *Sma* I (Takara, Japan), and the primers TEjAGL17F and TEjAGL17R. The vector expressing *EjAGL17* under the control of the CaMV 35S promoter was constructed and transformed into *Agrobacterium tumefaciens* strain GV3101-90. The 35S::*EjAGL17* was transformed into wild-type *Arabidopsis* using the floral-dip method [37]. Then, transgenic seeds were germinated on a solid 0.5×MS medium including 50 μg/ml kanamycin at 4°C for 2 days. These seeds after vernalization were transferred to the greenhouse under the conditions of 16 h light/8 h dark at 22°C for 2 weeks. Transgenic *Arabidopsis* lines were selected according to the described method [35]. Subsequently, the *EjAGL17* expression levels of transgenic lines were detected by qRT-PCR with the primers qEjAGL17F and qEjAGL17R, qagl17F and qagl17R [3]. As an internal control, the *Actin* of *Arabidopsis* was used with the primers qactinF and qactinR [38]. These primers of vectors construction and qRT-PCR were shown in S4 Table.

## 3. Results

### 3.1 Analysis of sequencing, assembly, annotation and DEGs

**Fig 1:**
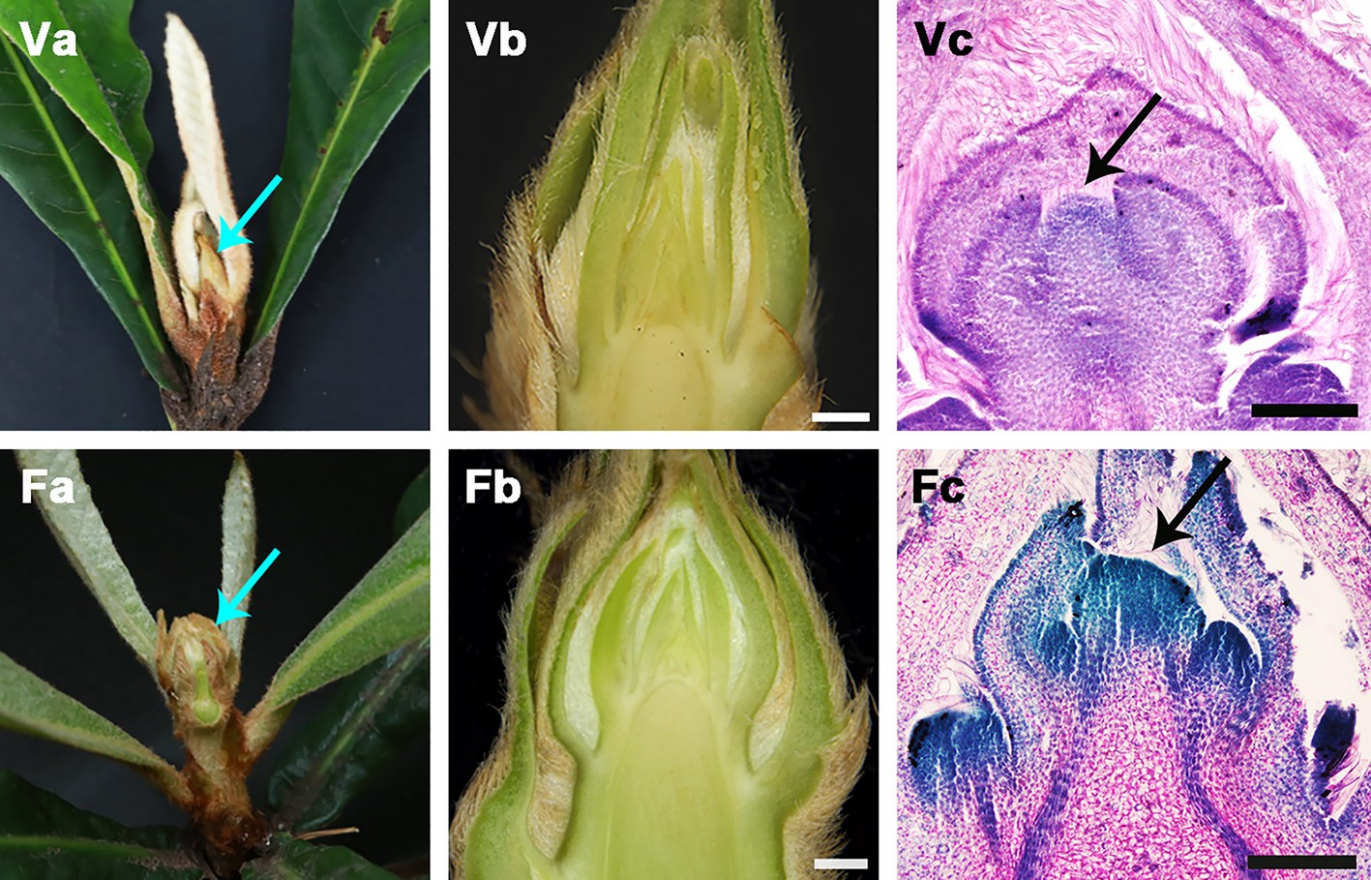
Flower bud transition in loquat. Va, vegetative apex (VA) was embraced by rudimentary leaves (blue arrow); Vb, morphological anatomy of VA; Vc, microscopic observations of VA (black arrow); Fa, flower bud transition (FBT) and the bud was swelled (blue arrow); Fb, morphological anatomy of FBT; Fc, microscopic observations of FBT (black arrow).

Developmental stages of vegetative apex (VA) and flower bud transition (FBT) were observed in loquat (Fig 1). The VA was embraced by rudimentary leaves. Then, FBT were formed from VA and the bud was swelled. The paraffin section observation of the apical buds showed that the transformation from vegetative to reproductive growth was from VA to FBT (Fig 1Vc and 1Fc).

**Fig 2:**
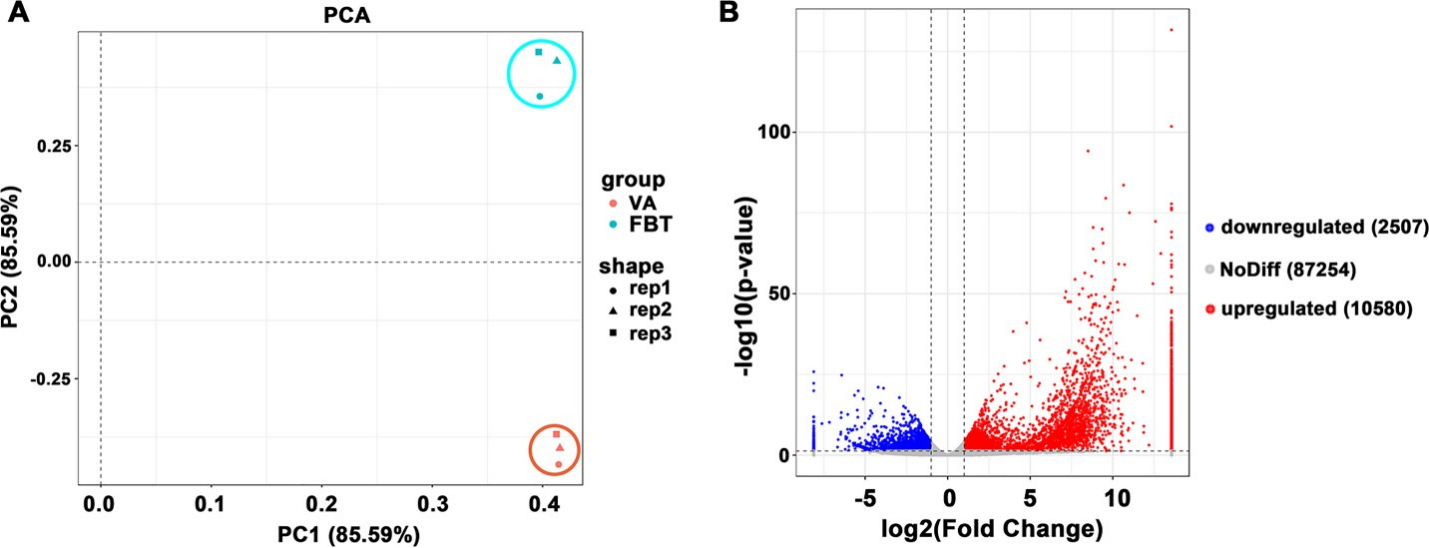
Analysis of gene expression cluster and DEGs numbers. (A) Principal components analysis of gene expression cluster. (B) Numbers of DEGs in FBT.

To identify candidate genes for the floral initiation, RNA-Seq libraries were constructed from VA and FBT. Over 6.1 Gb data of RNA sequencing from each sample was obtained with Q20 and Q30 values that were all greater than 90% (S5 Table). A total of 109 965 unigenes were generated with an average length of 837.1 bp and N50 of 1 527 bp (Table 1). Principal component analysis (PCA) analysis of global gene expression in VA and FBT were classified into two distinct groups, reflecting significant differences in global gene expression from VA to FBT. For each sample of the two developmental stages, three biological replicates were gathered into one cluster, suggesting the correlation of the biological replicates were high (Fig 2A).

**Table 1:** Summary of de novo assembly for VA and FBT.

	Transcript	Unigene
Total Length (bp)	374,265,354	92,048,617
Sequence Number	312,595	109,965
Mean Length (bp)	1197.3	837.1
N50 (bp)	1971	1527
N50 Sequence Number	61,659	17,263
N90 (bp)	522	309
N90 Sequence Number	196,950	72,704
GC %	42.94	43.91

In total, 52 750, 45 770, 41 679, 18 950 and 6045 unigenes were annotated in the NR, eggNOG, Swiss-Prot, GO and KEGG databases, respectively. In all five databases, 3379 (3.07%) unigenes were annotated (S1 Fig and S6 Table). Based on GO term analysis, the unigenes were classified into biological process, cellular component and molecular function (S2 Fig). The biological processes were mainly involved in metabolic process, cellular process and single-organism process. The cellular components were mainly cell, cell part and membrane. The molecular function was mainly involved in binding and catalytic activity. Meanwhile, the unigenes from the two stages were mapped into the pathways of metabolism, genetic information processing, environmental information processing, cellular processes and organismal systems on the basis of KEGG enrichment (S3 Fig).

### 3.2 Analysis of key DEGs involved in hormone signal transduction pathways

To obtain gene alterations in floral initiation, comparative transcriptome analysis of FBT vs VA were performed to detect floral initiation-related genes. In comparison of FBT vs VA, 13 087 DEGs were identified, of which 10 580 were upregulated and 2507 were downregulated (Fig 2B). The DEGs were mainly involved in plant hormone signal transduction (23 transcripts), carbon metabolism (29 transcripts), phenylpropanoid biosynthesis (17 transcripts), glyoxylate and dicarboxylate metabolism (13 transcripts) and nitrogen metabolism (12 transcripts) (S4 Fig).

**Fig 3:**
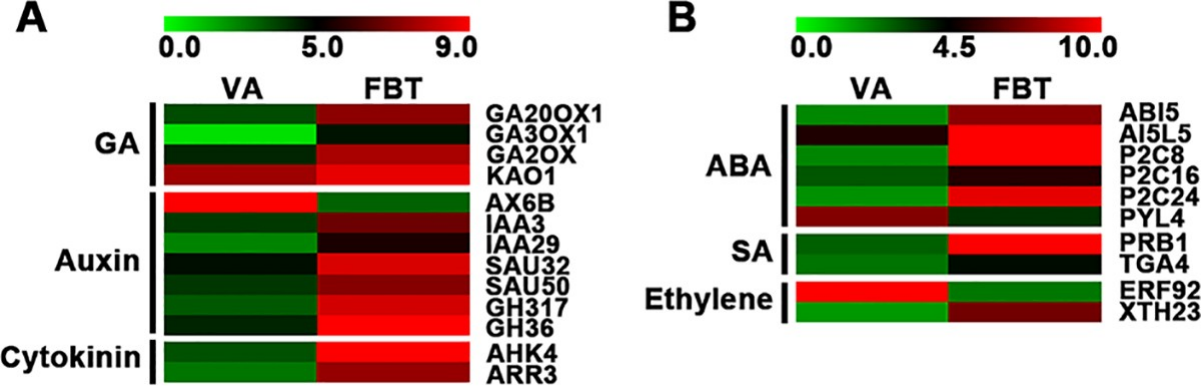
Expression analysis of DEGs involved in plant hormone signal transduction. (A) DEGs involved in the signaling pathways of GA, auxin and cytokinine. (B) DEGs involved in the signaling pathways of ABA, SA and ethylene.

To further investigate plant hormone signal transduction of the floral initiation, we analyzed gene expression of key DEGs in the signaling pathways of gibberellin (GA), auxin, cytokinine, abscisic acid (ABA), salicylic acid (SA) and ethylene (Fig 3, Tables 2 and 3). In the GA signaling pathway, a total of 4 key genes, including *GA20OX2*, *GA3OX1*, *GA2OX* and *KAO1*, were identified and all were significantly upregulated at FBT stage (Fig 3A and Table 2). In the auxin signaling pathway, a total of 7 key genes were identified, of which *IAA3*, *IAA29*, *SAUR32*, *SAUR50*, *GH317* and *GH36* were significantly upregulated at FBT stage (Fig 3A and Table 2). A total of 2 key genes, including *AHK4* and *ARR3*, were identified and significantly upregulated in cytokinin signaling pathway at FBT stage (Fig 3A and Table 2). In ABA signaling pathway, six key genes were identified, including *ABI5*, *AI5L5*, *PP2C8*, *PP2C16*, *PP2C24* and *PYL4*, of which *PYL4* was significantly downregulated (Fig 3B and Table 3). In SA signaling pathway, two genes, including *PRB1* and *TGA4*, were identified and both were significantly upregulated at FBT stage (Fig 3B and Table 3). A total of 2 key genes, including *ERF92* and *XTH23*, were identified in ethylene signaling pathways, but *ERF92* was significantly downregulated at FBT stage (Fig 3B and Table 3).

**Table 2:** DEGs involved in the signaling pathways of GA, auxin and cytokinin.

Gene_ID	Annotation	^a^ FC (FBT/VA)		*P* value
GA signaling pathway				
DN39821_c0_g1	Gibberellin 20 oxidase 2 (GA20OX2)	13.14	Up	7.7648×10^−9^
DN32181_c0_g1	Gibberellin 3-beta-dioxygenase 1 (GA3OX1)	16.36	Up	0.0045
DN42423_c2_g1	Gibberellin 2-beta-dioxygenase (GA2OX)	10.64	Up	3.2102×10^−6^
DN41605_c0_g2	Ent-kaurenoic acid oxidase 1 (KAO1)	2.23	Up	0.0002
Auxin signaling pathway				
DN42017_c1_g10	Auxin-induced protein 6B (AX6B)	0.12	Down	0.0088
DN24631_c0_g1	Auxin-responsive protein IAA3 (IAA3)	2.97	Up	0.0133
DN46222_c1_g2	Auxin-responsive protein IAA29 (IAA29)	6.52	Up	0.0005
DN35870_c1_g2	Auxin-responsive protein SAUR32 (SAUR32)	2.21	Up	0.0376
DN32884_c1_g2	Auxin-responsive protein SAUR50 (SAUR50)	3.08	Up	0.0097
DN31440_c0_g1	Indole-3-acetic acid-amido synthetase GH3.17 (GH317)	5.97	Up	3.2203×10^−5^
DN41385_c1_g1	Indole-3-acetic acid-amido synthetase GH3.6 (GH36)	2.03	Up	0.0004
Cytokinin signaling pathway				
DN45628_c1_g1	Histidine kinase 4 (AHK4)	2.06	Up	0.0122
DN39006_c2_g2	Two-component response regulator ARR3 (ARR3)	2.22	Up	4.5806×10^−5^

^a^ FC = Fold Change (FBT/VA). 0 ≤ FC≤ 0.5 indicates downregulation. FC ≥ 2 indicates downregulation. Up: upregulated unigenes. Down: downregulated unigenes. They are the same below.

**Table 3:** DEGs involved in the signaling pathways of ABA, SA and ethylene.

Gene_ID	Annotation	FC (FBT/VA)		*P* value
ABA signaling pathway				
DN43301_c1_g1	Abscisic acid-insensitive 5 (ABI5)	2.67	Up	8.1970×10^−5^
DN38203_c0_g4	Abscisic acid-insensitive 5-like protein 5 (AI5L5)	2.10	Up	0.0168
DN36087_c0_g1	Protein phosphatase 2C 8 (P2C8)	4.28	Up	5.6161×10^−14^
DN33780_c0_g2	Protein phosphatase 2C 16 (P2C16)	2.52	Up	0.0441
DN44811_c1_g1	Protein phosphatase 2C 24 (P2C24)	5.94	Up	1.4114×10^−7^
DN40379_c3_g2	Abscisic acid receptor PYL4 (PYL4)	0.42	Down	0.0011
SA signaling pathway				
DN27310_c0_g1	Pathogenesis-related protein 1 (PRB1)	8.21	Up	0.2647
DN26063_c0_g1	Transcription factor TGA4 (TGA4)	4.17	Up	0.0684
Ethylene signaling pathway				
DN28998_c0_g1	Ethylene-responsive transcription factor 1B (ERF92)	0.26	Down	3.7658×10^−10^
DN36915_c0_g3	Xyloglucan endotransglucosylase/hydrolase protein 23 (XTH23)	2.23	Up	0.0497

### 3.3 Identification of flower initiation-related transcription factors

**Fig 4:**
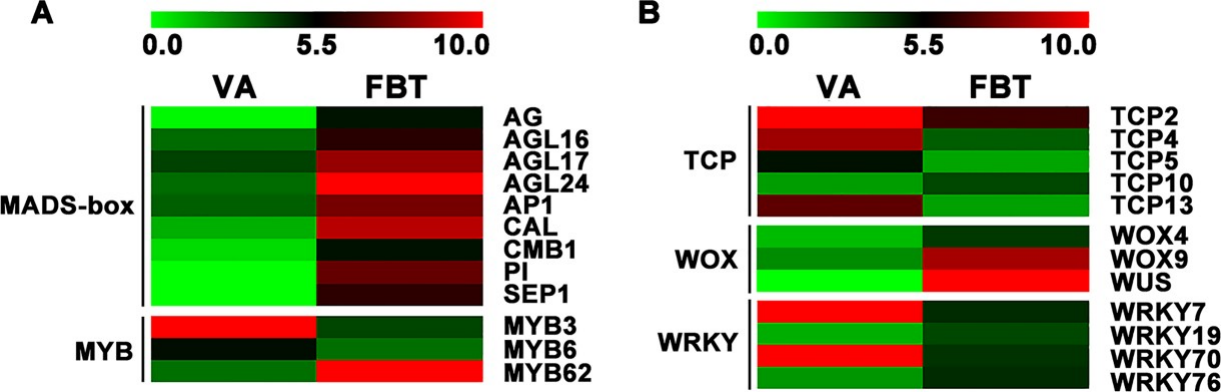
Analysis of key transcription factor genes in floral initiation.

Based on expression levels of key DEGs, we further analyzed the expression dynamics of key TF genes, such as *MADS-box*, *MYB* (Myeloblastosis), *TCP* (TEOSINTE BRANCHED 1, CYCLOIDEA and PCF1), *WOX* (WUSCHEL-related homeobox) and *WRKY* (WRKY DNA-binding protein) at flower bud transition stage. In *MADS-box* family genes, the expression levels of *AGAMOUS* (*AG*), *AGAMOUS-Like 16* (*AGL16*), *AGL17*, *AGL24*, *APETALA1* (*AP1*), *CAULIFLOWER* (*CAL*), *Carnation MADS Box 1* (*CMB1*), *PISTILLATA* (*PI*) and *SEPALLATA* (*SEP1*), were significantly upregulated (Fig 4A and Table 4). In *MYB* family genes, three key genes, including *MYB3*, *MYB6* and *MYB62* were identified, and expression levels of *MYB3* and *MYB6* were significantly downregulated (Fig 4A and Table 4). In *TCP* family genes, a total of 5 key genes were identified, of which *TCP2*, *TCP4*, *TCP5* and *TCP13* were significantly upregulated at flower bud transition stage (Fig 4B and Table 4). In *WOX* family genes, two genes, including *WOX4* and *WOX9*, were identified and both were significantly upregulated at FBT stage (Fig 4B and Table 4). In *WRKY* family genes, four key genes were identified, including *WRKY7*, *WRKY19*, *WRKY70* and *WRKY76*, of which *WRKY7* and *WRKY70* were significantly downregulated (Fig 4B and Table 4).

**Table 4:** The expression of key transcription factors in floral initiation.

Gene_ID	Annotation	FC (FBT/VA)		*P* value
MADS-box				
DN19033_c0_g1	Floral homeotic protein AGAMOUS (AG)	31.21	Up	0.0002
DN40991_c0_g1	Agamous-like MADS-box protein 16 (AGL16)	2.08	Up	0.0286
DN37075_c0_g1	Agamous-like MADS-box protein 17 (AGL17)	2.20	Up	0.0007
DN44520_c1_g3	AGL24 MADS-box protein 24 (AGL24)	4.24	Up	4.8571×10^−15^
DN41297_c0_g5	Floral homeotic protein APETALA 1 (AP1)	18.33	Up	0.0260
DN44752_c1_g4	Transcription factor CAULIFLOWER (CAL)	136.94	Up	1.6800×10^−49^
DN35997_c1_g2	MADS-box protein CMB1 (CMB1)	20.49	Up	0.0011
DN35997_c1_g3	Developmental protein SEPALLATA 1 (SEP1)	80.15	Up	0.0205
MYB				
DN40114_c3_g1	Transcription factor MYB3 (MYB3)	0.23	Down	0.0002
DN39018_c0_g1	Transcription factor MYB6 (MYB6)	0.23	Down	0.0056
DN39749_c3_g1	Transcription factor MYB62 (MYB62)	10.37	Up	1.897×10^−9^
TCP				
DN46671_c1_g1	Transcription factor TCP2 (TCP2)	0.41	Down	3.2263×10^−5^
DN36486_c1_g1	Transcription factor TCP4 (TCP4)	0.36	Down	0.0002
DN39943_c1_g1	Transcription factor TCP5 (TCP5)	0.24	Down	0.0012
DN27152_c0_g1	Transcription factor TCP10 (TCP10)	2.56	Up	0.0441
DN34930_c0_g1	Transcription factor TCP13 (TCP13)	0.34	Down	0.0078
WOX				
DN37723_c0_g1	WUSCHEL-related homeobox 4 (WOX4)	2.24	Up	0.0173
DN36324_c0_g1	WUSCHEL-related homeobox 9 (WOX9)	4.05	Up	4.3454×10^−5^
DN20573_c0_g1	Protein WUSCHEL (WUS)	86.47	Up	0.0063
WRKY				
DN38859_c0_g1	WRKY transcription factor 7 (WRKY7)	0.64	Down	0.0380
DN46827_c1_g3	WRKY transcription factor 19 (WRK19)	2.04	Up	0.0061
DN40348_c5_g1	WRKY transcription factor 70 (WRK70)	0.35	Down	0.0045
DN39683_c3_g1	WRKY transcription factor 76 (WRK76)	4.48	Up	0.0046

### 3.4 Validation of the expression analysis of several key flower initiation-related genes

**Fig 5:**
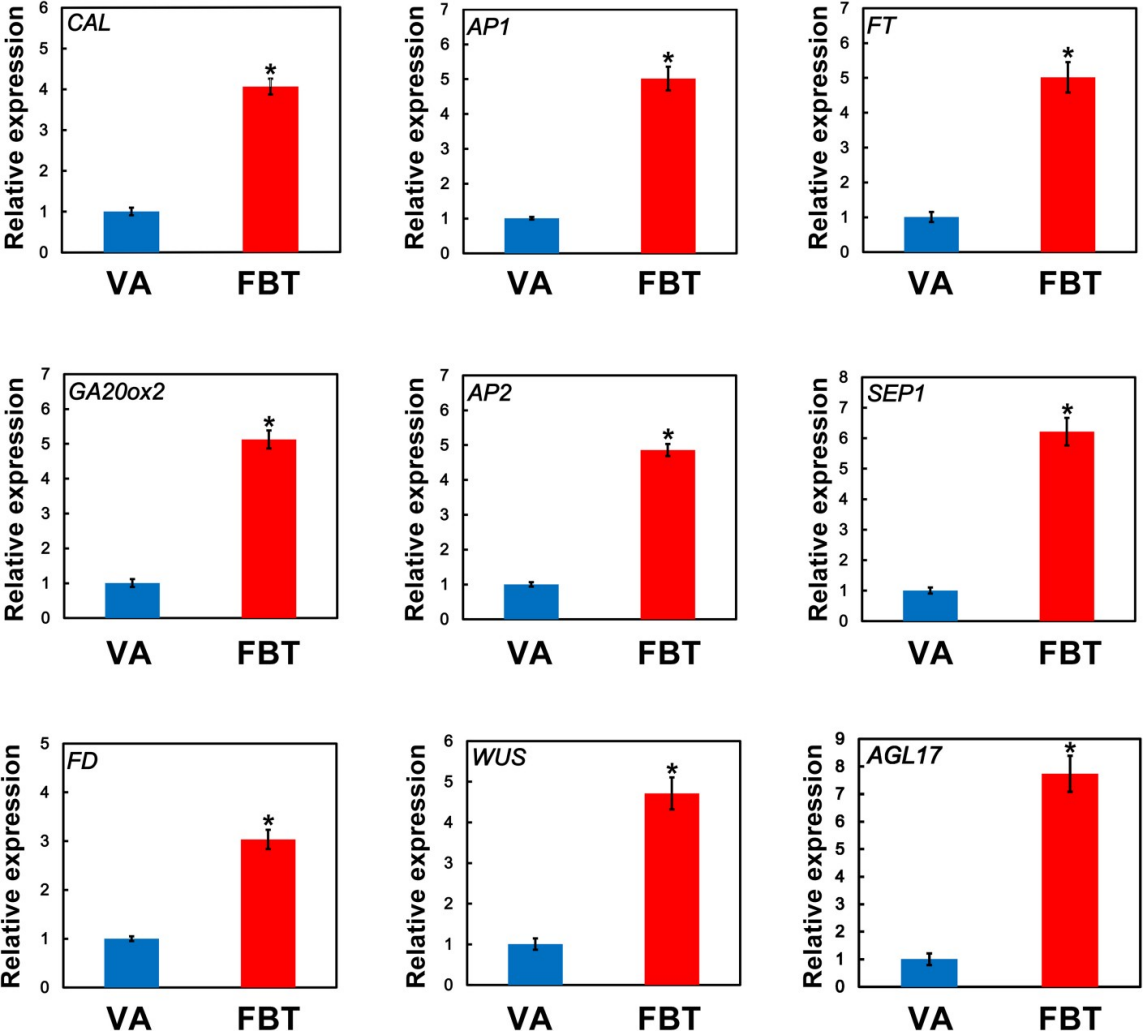
Validation of the expression of nine floral initiation-related genes by qRT-PCR analysis. Error bars indicate the standard deviation of three biological replicates. * is indicated significant difference at *P* < 0.05.

Analysis of qRT-PCR was used to validate the expression levels of several key DEGs that were identified using RNA-Seq. A total of nine flower bud transition-related unigenes were randomly detected via qRT-PCR (Fig 5). The expression levels of these nine genes corresponded well with the FPKM values obtained by RNAseq, suggesting that the correlation coefficients between the FPKM and qRT-PCR values were high.

### 3.5 Sequence and phylogenetic analysis of *EjAGL17*

**Fig 6:**
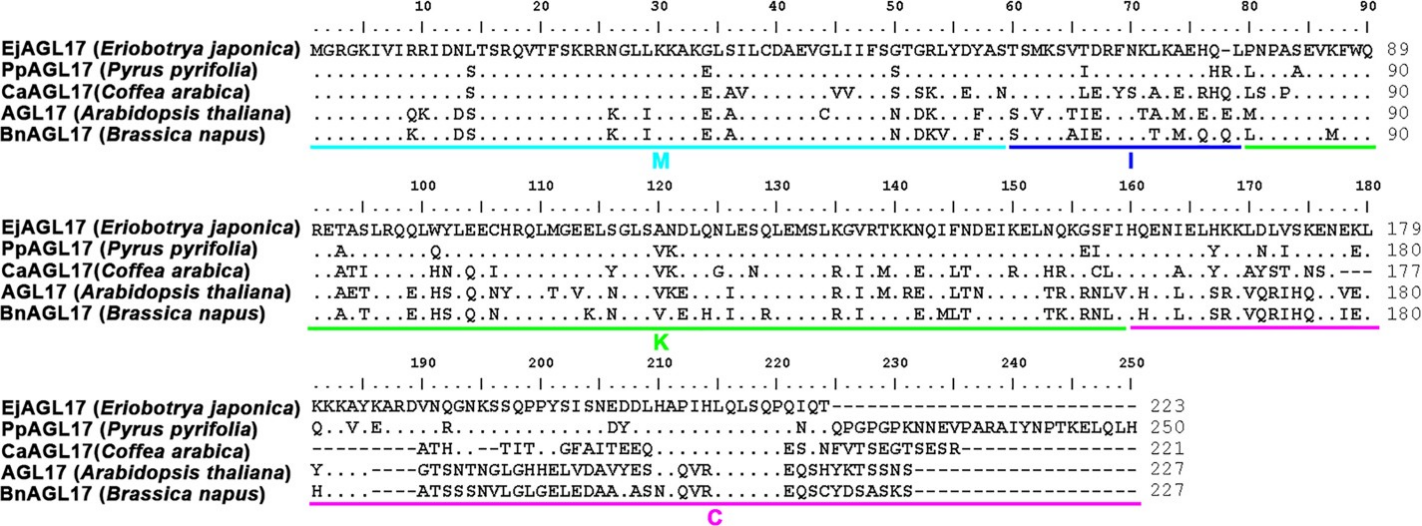
Analysis of sequence comparisons of EjAGL17. (A) Sequence comparisons of EjAGL17. The underlined regions represent the M, I, K and C domain. Amino acid residues identical to EjAGL17 are indicated as dots. To improve the alignment, dashes were introduced into the sequence.

Based on the analysis of key DEGs, one candidate gene *EjAGL17* (DN37075_c0_g1) was isolated from the MADS-box family. Comparative protein sequence analysis between EjAGL17 and other four AGL17 orthologous proteins showed that EjAGL17 has a 59-aa highly conserved MADS domain (1–59 aa), a 19-aa I domain (60–78 aa), an 80-aa K domain (79–158 aa) and a 65-aa completely divergent C domain (159–223 aa) (Fig 6). Meanwhile, these AGL17 orthologous proteins including EjAGL17 contained a distinctive C terminal domain (Fig 6). Molecular weight and theoretical isoelectric points of EjAGL17 protein are 25.59 kD and 9.48, respectively (S7 Table). Accession number of EjAGL17 sequence was MT344109 in the GenBank.

**Fig 7:**
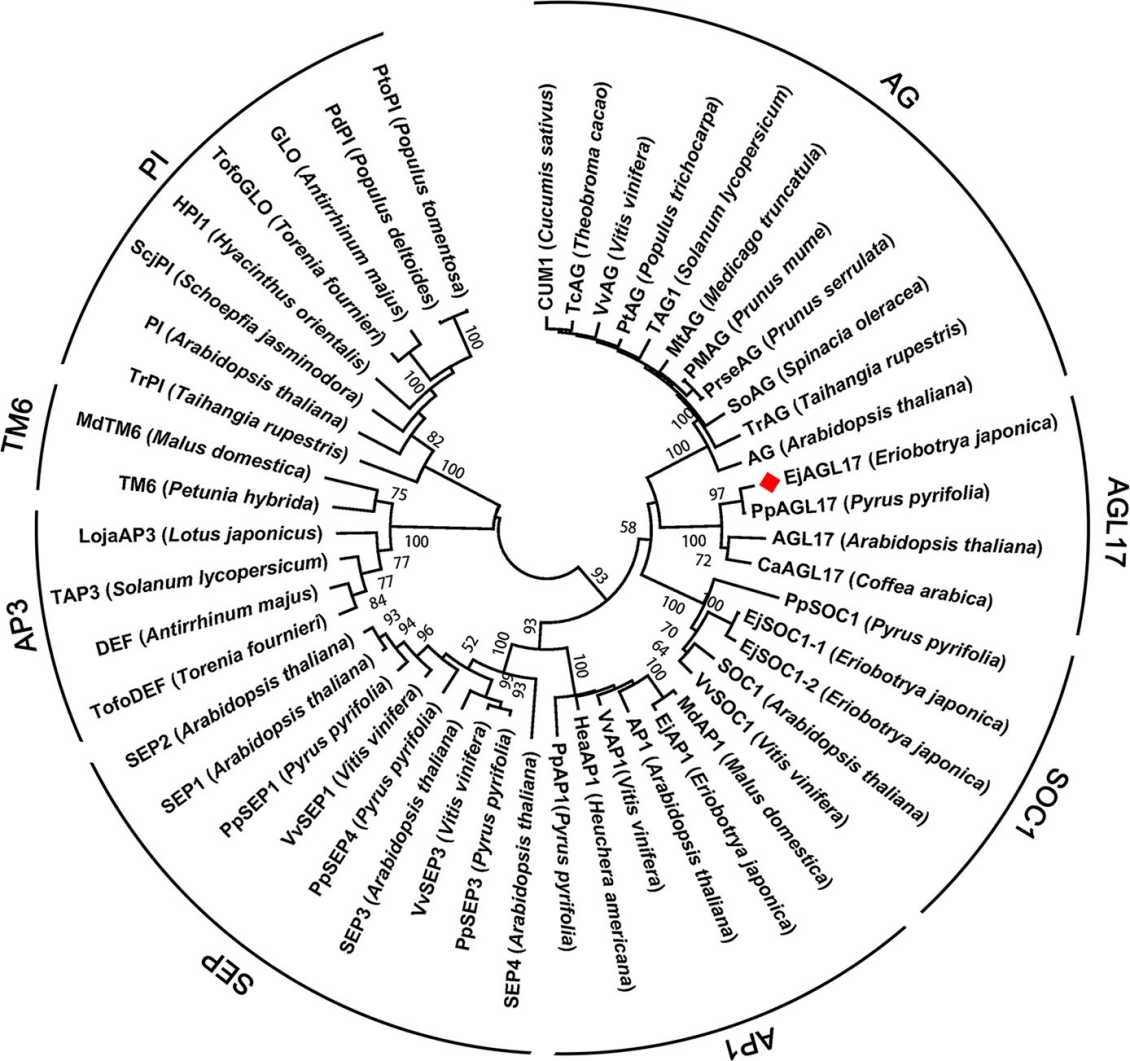
Phylogenetic analysis of MADS-box gene family. Protein sequence of EjAGL17 is blasted with eleven AG-clade genes, five SOC1-clade, six AP1-clade, nine SEP-clade, four AP3-clade, eight PI-clade and two TM6-clade proteins. EjAGL17 protein is marked.

Protein sequence of EjAGL17 was blasted to reveal the molecular evolution with AG, SOC1, AP1, SEP, AP3, TM6 and PI clade proteins from other angiosperms. Phylogenetic tree analysis revealed that the *EjAGL17* is grouped into the AGL17-clade of MADS-box transcription factors in the eudicots (Fig 7).

### 3.6 Subcellular localization and expression levels of *EjAGL17*

**Fig 8:**
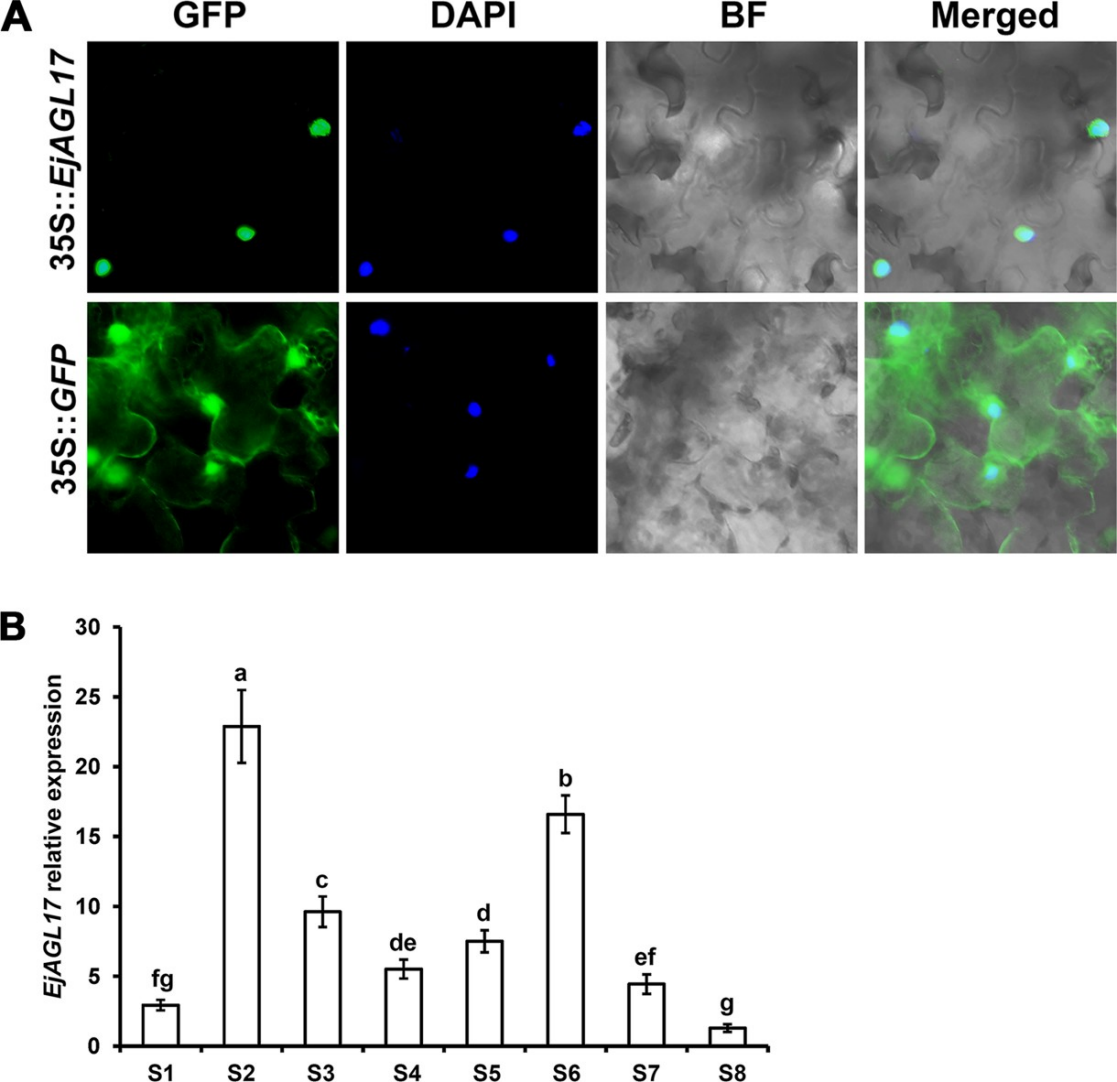
Subcellular localization and relative expression of *EjAGL17* in flower development of early and late flowering cultivars in loquat at the stages of S1–S8. (A) Subcellular localization of *EjAGL17*. GFP, green fluorescent protein; DAPI, 4,6-diamidino-2-phenylindole; BF, bright-field; Merged, merged image of GFP, DAPI and BF. (B) Relative expression of *EjAGL17*.

Subcellular localization of 35S::*EjAGL17-GFP* were detected and transiently expressed in epidermal cells of *N*. *benthamiana* leaf. As a negative control, fluorescence of the 35S::*GFP* was observed in both the nucleus and cytoplasm. However, fluorescence of 35S::*EjAGL17-GFP* was found only in the nucleus (Fig 8A). These results showed that EjAGL17 is a nuclear-localized protein and its subcellular localization pattern is consistent with that of *AGL17* orthologs in soybean and maize [39, 40], having subcellular localization characteristics of typical transcription factors.

During the process of flower development, expression of *EjAGL17* was persisted from vegetative apex to full bloom in loquat. Meanwhile, the expression levels of *EjAGL17* reached the highest at the development stage of FBT (Fig 8B).

### 3.7 Ectopic expression of *EjAGL17* in *Arabidopsis*

**Fig 9:**
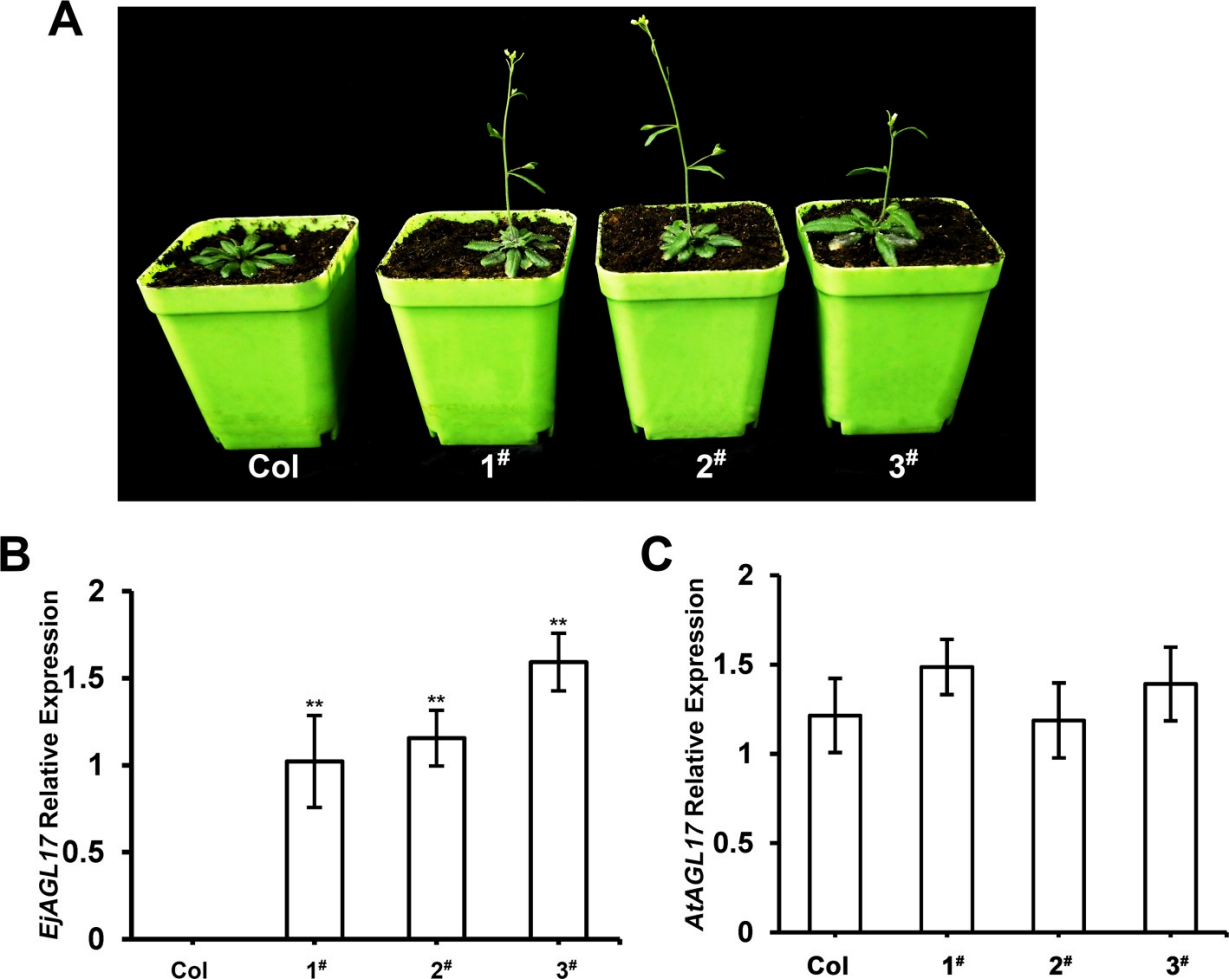
Comparison of the wild-type and 35S::*EjAGL17* transgenic *Arabidopsis*. (A) 35S::*EjAGL17* transgenic *Arabidopsis* significantly promoted early flowering. Col, wild-type *Arabidopsis*; line 1^#^-3^#^, 35S::*EjAGL17* transgenic wild-type *Arabidopsis*. (B) Expression levels of *EjAGL17* in transgenic wild-type lines were significantly higher than that in untransgenic wild-type *Arabidopsis*. (C) Expression levels of the *AtAGL17* were no significant difference between transgenic and untransgenic wild-type *Arabidopsis*. ** indicates significantly different at *P* < 0.01.

To further investigate the function of *EjAGL17*, overexpression assays were conducted by ectopic expression of *EjAGL17* in *Arabidopsis*. A total of thirty-five 35S::*EjAGL17* transgenic *Arabidopsis* lines were obtained by screening and detecting with MS medium containing kanamycin. Flowering phenotypes of three T3 generation of 35S::*EjAGL17* transgenic lines were observed. Comparison with untransformed lines, 35S::*EjAGL17* transgenic plants caused early flowering under the same long-day conditions (Fig 9A).

Expression levels of *EjAGL17* in transgenic lines were significantly higher than that of untransgenic *Arabidopsis* (Fig 9B). However, expression levels of *Arabidopsis AtAGL17* gene were no significant difference between transgenic and untransgenic *Arabidopsis* (Fig 9C).

## 4. Discussion

Floral initiation of woody angiosperm undergoes morphological changes, and is generally controlled by the regulatory network and environmental signals [41, 42]. However, reproductive development of loquat is a continuous process, and do not exhibit dormancy [18, 19], indicating that expression pattern of genes controlling floral initiation might be distinctive. In this study, we used comparative transcriptome analysis to investigate gene expression changes at the stages of VA and FBT. The DEGs were mainly involved in the hormone signal transduction pathways and transcription factor genes. Among these DEGs, we identified that one candidate gene *EjAGL17* was involved in floral initiation. Our study provides abundant candidate genes for studying the mechanisms underlying the floral initiation in loquat.

### 4.1 Expression pattern of key DEGs associated with floral initiation

In our study, the DEGs were mainly involved in the signaling pathways of GA, auxin, cytokinin, ABA, SA and ethylene. Previously, in *Litchi chinensis*, comparative transcriptomic analysis of floral initiation was mainly enriched in the metabolic pathways of plant hormone signal transduction, and the DEGs were mainly involved in the biosynthesis of auxin, cytokinin, jasmonic acid (JA), SA and ABA [43]. In *Camellia sinensis*, transcriptomic analysis of flower development indicated that DEGs were mainly associated with metabolic pathways of auxin, cytokinin, GA, ABA, ethylene, brassinosteroid, JA and SA [44]. In *Malus domestica*, transcriptomic analysis of flower induction showed that the DEGs were mainly enriched in cytokinin, ABA and GA pathways [45]. However, transcriptomic analysis of floral bud initiation, floral organ differentiation and bud outgrowth revealed that DEGs were mainly involved in the signaling pathways of GA, auxin, JA and brassinosteroid in *Camellia azalea* [42]. In *Annona squamosa*, comparative transcriptomic analysis of floral transition and flower development reported that DEGs were mainly enriched in the signaling pathways of GA, auxin, cytokinin and ABA [46]. These previous studies and our data suggest that key DEGs are mainly enriched in hormone signal transduction pathways in floral initiation.

### 4.2 DEGs of key transcription factors that affect floral initiation

In our work, transcription factors, including *MADS-box*, *MYB*, *TCP*, *WOX* and *WRKY*, were significantly differentially expressed at FBT stage. Similarly, some transcription factors, such as *MADS-box*, *TCP* and *MYB*, were also significantly differentially expressed at flower development in double-flower loquat [28]. Especially, MADS-box transcription factors play important regulatory roles in floral transition, flower organ identity determination and flowering time control in the molecular networks in angiosperm [5–7]. Importantly, MIKC-type MADS-domain transcription factors exhibit a characteristic domain organization and contain a MADS (M), an intervening (I), a keratin-like (K) and a C-terminal (C) domain [47–49]. The designated domains of MADS-domain transcription factors provide a fundament for studying gene regulation of flower development in angiosperms [50].

### 4.3 Expression pattern and functional analysis of *EjAGL17*

In our study, *EjAGL17* expression level were upregulated at the stage of floral initiation in loquat. Meanwhile, expression pattern of *EjAGL17* is responsible largely for its functional specificity, and is a good predictor of function. This indicated that *EjAGL17* might be associated with floral initiation in loquat. Previously, *PpAGL17* were mainly detected in the flower in *P*. *pyrifolia* [51]. However, expression level of *AGL17* ortholog in soybean was much higher in roots and nodules than that in other organs [39]. In rice, *AGL17* ortholog was mainly expressed in root development [52, 53]. In maize, *AGL17* ortholog was mainly expressed in root xylem parenchyma cells [40]. These data showed that expression patterns of *AGL17* orthologs exhibit diverse in development process in angiosperm.

Ectopic expression of *EjAGL17* in *Arabidopsis* promoted early flowering, indicating function of *EjAGL17* is involved in flowering time in loquat. Meanwhile, expression levels of *EjAGL17* and *AtAGL17* were analyzed in transgenic *Arabidopsis* to verify that the early-flowering phenotype was caused by overexpression of *EjAGL17*. Similarly, overexpression of *AGL17* promoted early flowering, but mutant of *AGL17* caused late flowering in *Arabidopsis* [3, 4]. Therefore, we conclude that the functions of *AGL17* orthologs in regulating flowering time between loquat and *Arabidopsis* were conserved. However, in soybean, overexpression of *AGL17* orthologs in transgenic soybean significantly promotes the formation and development of lateral root [39]. Function of *AGL17* ortholog is involved in the regulation of root and seed development in rice, and participated in responses to osmotic stress and nutrient supply [52–54]. In maize, functional analysis of *AGL17* ortholog suggests that its involvement in root nitrate-foraging [40]. These data revealed that characterization of *AGL17* orthologs is involved in regulating different development aspects and functional diversification. Furthermore, function of *EjAGL17* should be further explored to reveal the possible roles in the control of other developmental programs. Our results provide a better understanding for the functional roles of *AGL17* orthologs involving in floral induction in *Eriobotrya*.

## 5. Conclusions

In this study, transcriptome expression and functional analyses were used to analyze gene expression changes at the stages of VA and FBT. The DEGs were mainly involved in the hormone signal transduction pathways. Meanwhile, some transcription factors were significantly differentially expressed. Among these DEGs, one candidate gene *EjAGL17*, which was significantly up-regulated at the development stage of floral initiation, was isolated and identified from loquat. Analyses of protein sequence and phylogenetic tree revealed that *EjAGL17* was grouped into the AGL17 clade MADS-box transcription factors, and included a distinctive C-terminal domain. Ectopic expression of *EjAGL17* transgenic *Arabidopsis* significantly promoted early flowering. Furthermore, other key DEGs should be further explored to reveal their possible roles in floral initiation. Our results provide a better understanding for metabolic pathways and candidate genes involving in floral induction in *Eriobotrya*.

## Supporting information

S1 Fig Venn diagram analysis of the unigenes annotated among five databases. (DOCX)

S2 Fig Gene Ontology analysis of assembled unigenes. (DOCX)

S3 Fig KEGG enrichment analysis of assembled unigenes. (DOCX)

S4 Fig KEGG pathway enrichment analysis of DEGs. (DOCX)

S1 Table Primer sequences used for qRT-PCR. (DOCX)

S2 Table Isolation of *EjAGL17* gene using the primer sequences. (DOCX)

S3 Table *EjAGL17* and multiple AG, AGL17, SOC1, AP1, SEP, AP3, PI and TM6-clade proteins proteins from various angiosperm lineages were selected, including their family names and the accession numbers. (DOCX)

S4 Table The primer sequences of subcellular localization and qRT-PCR of EjAGL17 gene. (DOCX)

S5 Table The RNA sequencing quality of egetative apex (VA) and flower bud transition (FBT) in loquat. (DOCX)

S6 Table Functional annotation for VA and FBT. (DOCX)

S7 Table Molecular weight and isoelectric points of EjAGL17 protein. (DOCX)
