# Supplementary figures and images for: Comparative transcriptome analysis of flower bud transition and functional characterization of EjAGL17 involved in regulating floral initiation in loquat
Source: PLoS One. 2020 Oct 8;15(10):e0239382. doi: 10.1371/journal.pone.0239382 (PMC7544058; doi:10.1371/journal.pone.0239382)

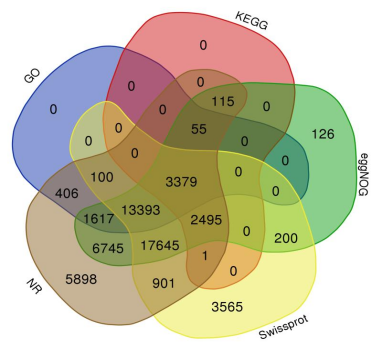


**Figure S1.** Venn diagram analysis of the annotated unigenes among five databases.

Supplement: S1 Fig — (DOCX) [file pone.0239382.s001.docx]

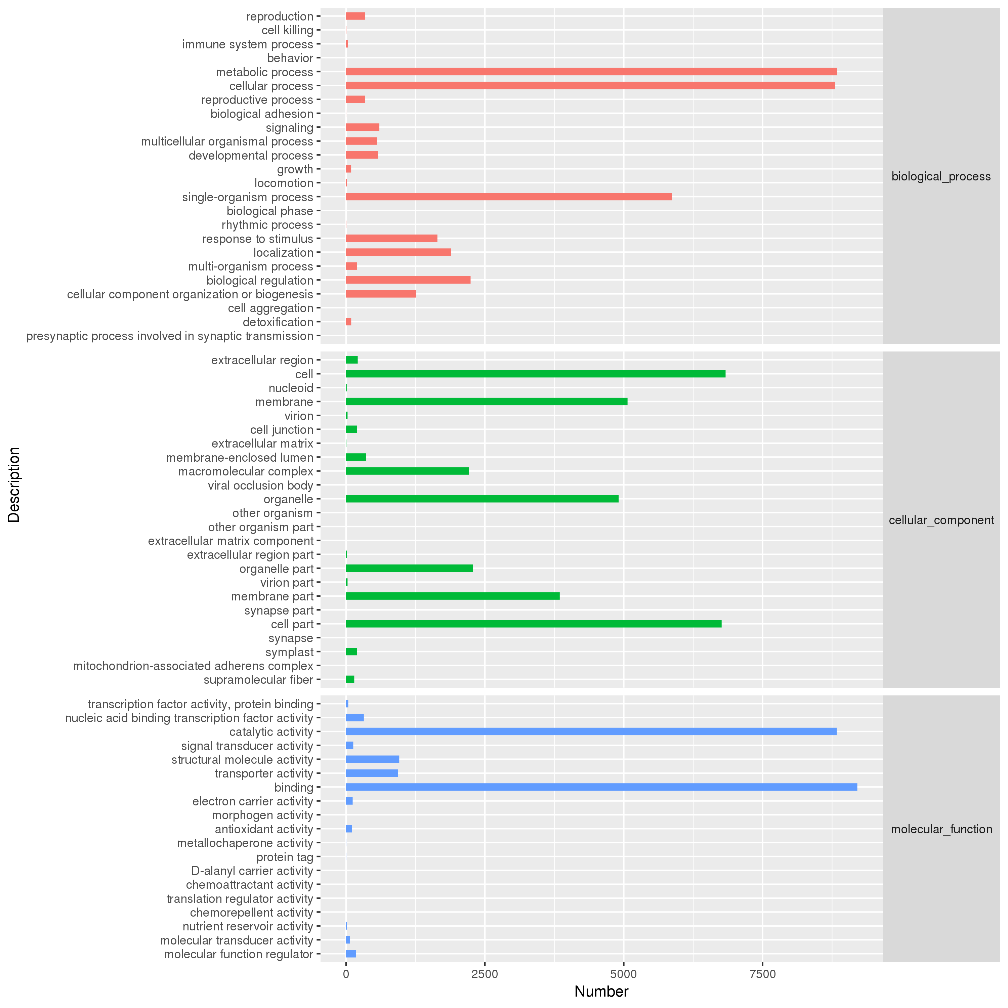


**Figure S2.** Gene Ontology analysis of assembled unigenes.

Supplement: S2 Fig — (DOCX) [file pone.0239382.s002.docx]

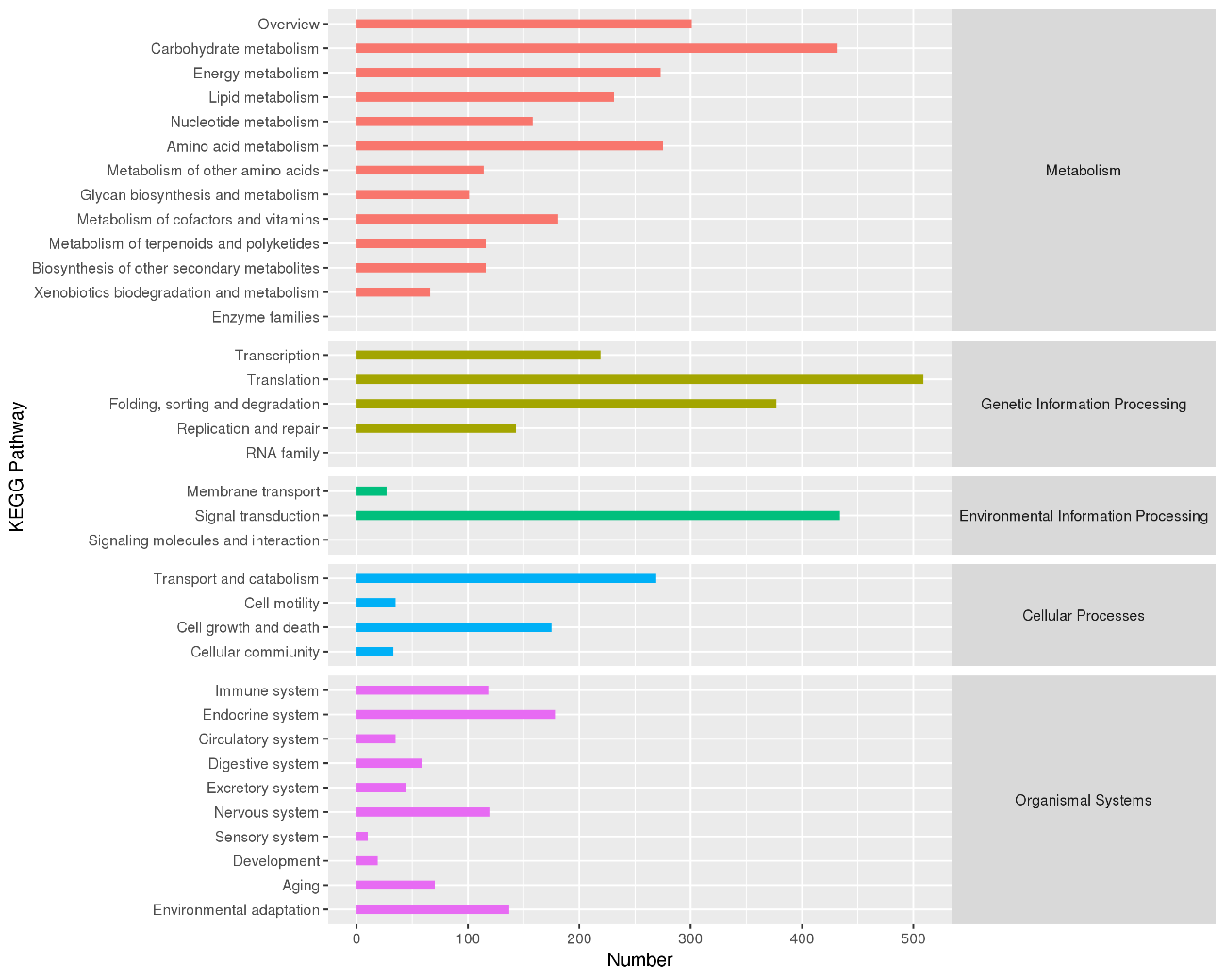


**Figure S3.** KEGG enrichment analysis of assembled unigenes.

Supplement: S3 Fig — (DOCX) [file pone.0239382.s003.docx]

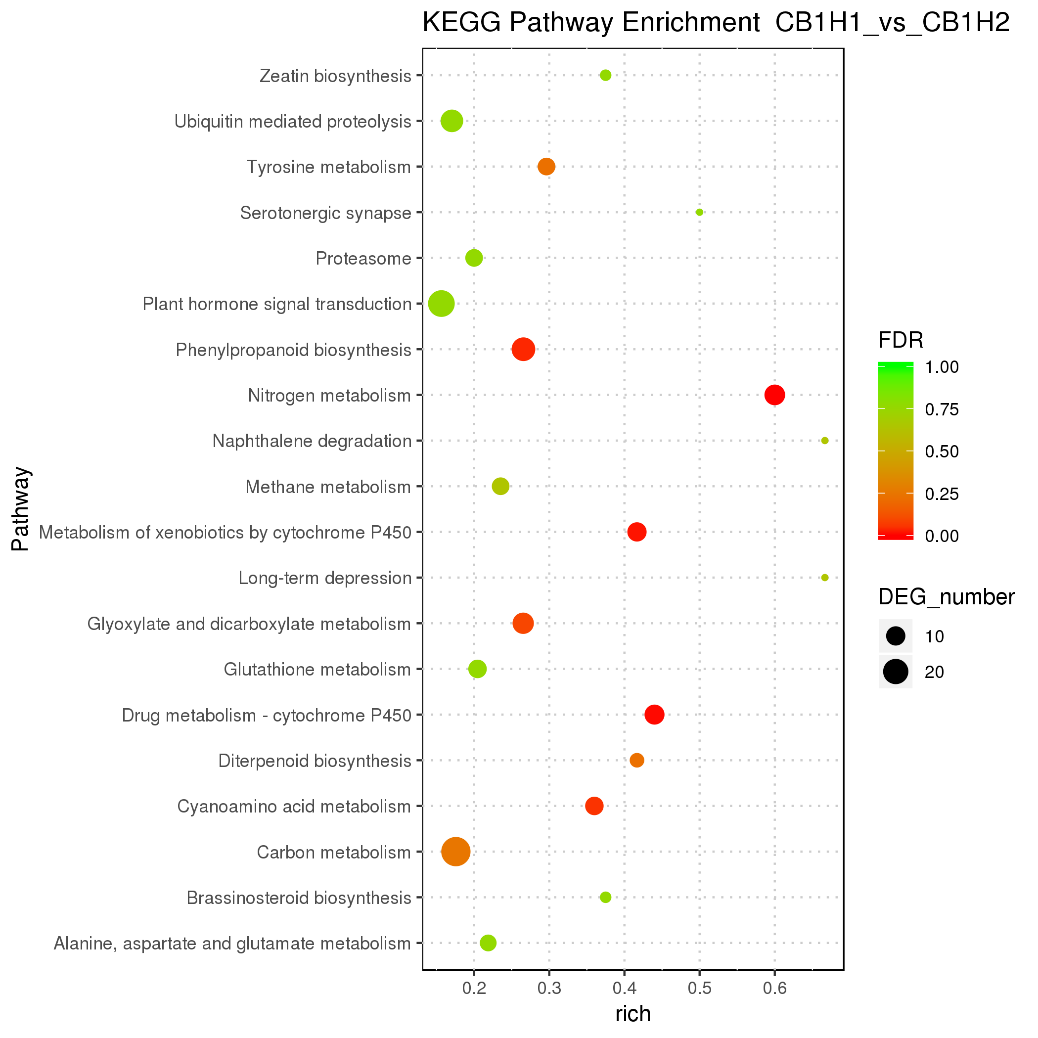


**Figure S4.** KEGG pathway enrichment analysis of DEGs.

Supplement: S4 Fig — (DOCX) [file pone.0239382.s004.docx]
