# Supplementary material for: Comparative transcriptome analysis of flower bud transition and functional characterization of EjAGL17 involved in regulating floral initiation in loquat
Source: PLoS One. 2020 Oct 8;15(10):e0239382. doi: 10.1371/journal.pone.0239382 (PMC7544058; doi:10.1371/journal.pone.0239382)
Supplement: S1 Table — (DOCX) [file pone.0239382.s005.docx]

Table S1. Primer sequences used for qRT-PCR.

| Gene | Primer | Primer sequences (5 to 3) |
| --- | --- | --- |
| Ejactin | EjactinF | AATGGAACTGGAATGGTCAAGGC |
|  | EjactinR | TGCCAGATCTTCTCCATGTCATCCCA |
| CAL | CALF | AACTACAACAACAACTGCAGC |
|  | CALR | TTACATCTTGTGGCCCTACAT |
| AP1 | AP1F | GCAGGGTAACTGGACCTTTG |
|  | AP1R | GCAGTGTCAAGCTGTTGCTCC |
| FT | FTF | GTTGTTGGACGAGTGGTAG |
|  | FTR | TAACCTCTTTATTGCCGTAG |
| GA20OX2 | GA20ox2F | AACCATGGCAAAACCTCCCT |
|  | GA20ox2R | GAGTGGGACTCGAAGCTCTG |
| AP2 | AP2F | GACGGTCGGAGGACCATCCAGC |
|  | AP2R | CGATAAAACGTAACGCCCCGAT |
| SEP1 | SEP1F | ATGGGGAGAGGAAGAGTGGAG |
|  | SEP1R | ATAGAGCTTGCCACGGTTGG |
| FD | FDF | CCAACACTTCTCCGCCTGAA |
|  | FDR | CAAAAGACGGCAAGCCGAAG |
| WUS | WUSF | TGGCAAGATCGAAGGCAAGAA |
|  | WUSR | TCAATCCCAACCCCTGATGA |
| AGL17 | AGL17F | TTGACGAGCAGGCAAGTGAC |
|  | AGL17R | TAGCATAATCGTAAAGCCTG |
