# Supplementary material for: Comparative transcriptome analysis of flower bud transition and functional characterization of EjAGL17 involved in regulating floral initiation in loquat
Source: PLoS One. 2020 Oct 8;15(10):e0239382. doi: 10.1371/journal.pone.0239382 (PMC7544058; doi:10.1371/journal.pone.0239382)
Supplement: S2 Table — (DOCX) [file pone.0239382.s006.docx]

Table S2 Isolation of *EjAGL17* gene using the primer sequences.

| Primer | Primer sequences (5′ to 3′) |
| --- | --- |
| 3RAGL17F1 | GAAGGCTAAGGGGCTATCAATCC |
| 3′RACE Outer Primer | TACCGTCGTTCCACTAGTGATTT |
| 3RAGL17F2 | GCGGAGCATCAACTGCCGAATCCG |
| 3′RACE Inner Primer | CGCGGATCCTCCACTAGTGATTTCACTATAGG |
| 5RAGL17R1 | CTTCTCATTCTCTTTACTTACGAGGT |
| 5RAGL17R2 | TATTTCATCATTAAATATCTGGTTC |
| 5′RACE Universal Primer Mix | TAATACGACTCACTATAGGGCAAGCAGTGGTATCAACGCAGAGT |
|  | CTAATACGACTCACTATAGGGC |
| FLAGL17F | ATGGGGAGAGGAAAGATTGTGATTAGA |
| FLAGL17R | ATTGTTCGAAGGCCCAAGCCCATG |
