# Supplementary material for: Comparative transcriptome analysis of flower bud transition and functional characterization of EjAGL17 involved in regulating floral initiation in loquat
Source: PLoS One. 2020 Oct 8;15(10):e0239382. doi: 10.1371/journal.pone.0239382 (PMC7544058; doi:10.1371/journal.pone.0239382)
Supplement: S3 Table — (DOCX) [file pone.0239382.s007.docx]

Table S3 *EjAGL17* and multiple AG, AGL17, SOC1, AP1, SEP, AP3, PI and TM6-clade proteins proteins from various angiosperm lineages were selected, including their family names and the accession numbers.

| Protein | Species | Accession number |
| --- | --- | --- |
| AG | *A. thaliana* | P17839.2 |
| CUM1 | *C. sativus* | NP_001292633.1 |
| TcAG | *T. cacao* | ABA39727.1 |
| VvAG | *V. vinifera* | ACZ26525.1 |
| PtAG | *P. trichocarpa* | AAC06237.1 |
| TAG1 | *S. lycopersicum* | AAA34197.1 |
| MtAG | *M. truncatula* | AIT11837.1 |
| PMAG | *P. mume* | ABU41518.1 |
| PrseAG | *P. serrulata* | ADK95058.1 |
| SoAG | *S. oleracea* | AAT91060.1 |
| TrAG | *G. rupestre* | ABB59994.1 |
| PpAGL17 | *P. pyrifolia* | AJW29043.1 |
| AGL17 | *A. thaliana* | OAP11731.1 |
| CaAGL17 | *C. arabica* | ADU56830.1 |
| PpSOC1 | *P. pyrifolia* | AJW29027.1 |
| EjSOC1-1 | *E. japonica* | QBQ57658.1 |
| EjSOC1-2 | *E. japonica* | QBQ57659.1 |
| SOC1 | *A. thaliana* | OAP11266.1 |
| VvSOC1 | *V. vinifera* | RVW41170.1 |
| MdAP1 | *M. domestica* | ACD69426.1 |
| EjAP1 | *E. japonica* | AAX14151.1 |
| AP1 | *A. thaliana* | CAA78909.1 |
| VvAP1 | *V. vinifera* | Q6E6S7.1 |
| HeaAP1 | *H. americana* | AAP83372.1 |
| PpAP1 | *P. pyrifolia* | AJW29044.1 |
| SEP1 | *A. thaliana* | NP_001119230.1 |
| SEP2 | *A. thaliana* | NP_186880.1 |
| SEP3 | *A. thaliana* | NP_850953.1 |
| SEP4 | *A. thaliana* | NP_849930.1 |
| VvSEP1 | *V. vinifera* | Q8LLR2.2 |
| VvSEP3 | *V. vinifera* | Q8LLR0.2 |
| PpSEP1 | *P. pyrifolia* | AJW29023.1 |
| PpSEP3 | *P. pyrifolia* | AJW29021.1 |
| PpSEP4 | *P. pyrifolia* | AJW29039.1 |
| LjAP3 | *L. japonicus* | AAX13301.1 |
| TAP3 | *S. lycopersicum* | CAJ53871.1 |
| DEF | *A. majus* | BAI68389.1 |
| TofoDEF | *T. fournieri* | BAG24492.1 |
| TM6 | *Petunia*×*hybrid* | AAS46017.1 |
| MdTM6 | *M. domestica* | BAC11907.1 |
| PtoPI | *P. tomentosa* | AGL09298.1 |
| PdPI | *P. deltoides* | ABS71831.1 |
| GLO | *A. majus* | CAA48725.1 |
| TofoGLO | *T. fournieri* | BAJ15423.1 |
| HPI1 | *H. orientalis* | AAD22493.2 |
| ScjPI | *S. jasminodora* | AFV74899.1 |
| PI | *A. thaliana* | NP_197524.1 |
| TrPI | *T. rupestris* | ABB59993.1 |
