# Supplementary material for: Comparative transcriptome analysis of flower bud transition and functional characterization of EjAGL17 involved in regulating floral initiation in loquat
Source: PLoS One. 2020 Oct 8;15(10):e0239382. doi: 10.1371/journal.pone.0239382 (PMC7544058; doi:10.1371/journal.pone.0239382)
Supplement: S4 Table — (DOCX) [file pone.0239382.s008.docx]

Table S4 The primer sequences of subcellular localization and qRT-PCR of *EjAGL17* gene.

| Primer | Primer sequences (5′ to 3′) |
| --- | --- |
| SLEjAGL17F | cgagctcATGGGGAGAGGAAAGATTGTGAT |
| SLEjAGL17R | cgggatccCTAAGTTTGAATCTGTGGCTGGC |
| qEjactinF | AATGGAACTGGAATGGTCAAGGC |
| qEjactinR | TGCCAGATCTTCTCCATGTCATCCCA |
| TEjAGL17F | TCTAGAATGGGGAGAGGAAAGATTGTGATTAG |
| TEjAGL17R | CCCGGGCTAAGTTTGAATCTGT GGCTGGC |
| qactinF | CGTATGAGCAAGGAGATCAC |
| qactinR | CACATCTGTTGGAAGGTGCT |
| qagl17F | GACATCTTCAAACAGCTAAGATCA |
| qagl17R | CGAGAAGCTGTTCCATTGC |
