# Supplementary material for: Comparative transcriptome analysis of flower bud transition and functional characterization of EjAGL17 involved in regulating floral initiation in loquat
Source: PLoS One. 2020 Oct 8;15(10):e0239382. doi: 10.1371/journal.pone.0239382 (PMC7544058; doi:10.1371/journal.pone.0239382)
Supplement: S5 Table — (DOCX) [file pone.0239382.s009.docx]

Table S5 The RNA sequencing quality of egetative apex (VA) and flower bud transition (FBT) in loquat.

| Sample | Total nucleotide (nt) | Clean Reads No. | Clean Reads (%) | Q20 (%) | Q30 (%) |
| --- | --- | --- | --- | --- | --- |
| VA-1 | 7,990,688,366 | 52,495,616 | 99.20 | 96.38 | 91.49 |
| VA-2 | 6,829,974,016 | 44,810,918 | 99.06 | 96.13 | 90.96 |
| VA-3 | 6,101,063,192 | 40,049,184 | 99.12 | 96.16 | 91.01 |
| FBT-1 | 7,081,198,800 | 46,627,462 | 98.77 | 96.37 | 91.42 |
| FBT-2 | 6,836,604,900 | 45,215,298 | 99.20 | 96.88 | 92.36 |
| FBT-3 | 7,320,533,100 | 48,411,136 | 99.19 | 96.87 | 92.30 |
