# Supplementary material for: Comparative transcriptome analysis of flower bud transition and functional characterization of EjAGL17 involved in regulating floral initiation in loquat
Source: PLoS One. 2020 Oct 8;15(10):e0239382. doi: 10.1371/journal.pone.0239382 (PMC7544058; doi:10.1371/journal.pone.0239382)
Supplement: S6 Table — (DOCX) [file pone.0239382.s010.docx]

Table S6 Functional annotation for VA and FBT.

| Database | Number | Percentage (%) |
| --- | --- | --- |
| NR | 52,750 | 47.97 |
| GO | 18,950 | 17.23 |
| KEGG | 6045 | 5.50 |
| eggNOG | 45,770 | 41.62 |
| Swissprot | 41,679 | 37.90 |
| In all database | 3379 | 3.07 |
