# Supplementary material for: Comparative transcriptome analysis of flower bud transition and functional characterization of EjAGL17 involved in regulating floral initiation in loquat
Source: PLoS One. 2020 Oct 8;15(10):e0239382. doi: 10.1371/journal.pone.0239382 (PMC7544058; doi:10.1371/journal.pone.0239382)
Supplement: S7 Table — (DOCX) [file pone.0239382.s011.docx]

Table S7 Molecular weight and isoelectric points of EjAGL17 protein.

| Protein | Molecular weight | Theoretical isoelectric points |
| --- | --- | --- |
| EjAGL17 | 25.59 kD | 9.48 |
